# Pelagic distribution of plastic debris (> 500 µm) and marine organisms in the upper layer of the North Atlantic Ocean

**DOI:** 10.1038/s41598-022-17742-7

**Published:** 2022-08-11

**Authors:** Matthias Egger, Britte Schilt, Helen Wolter, Thomas Mani, Robin de Vries, Erik Zettler, Helge Niemann

**Affiliations:** 1 The Ocean Cleanup, Rotterdam, The Netherlands; 2 Egger Research and Consulting, St. Gallen, Switzerland; 3 Department of Earth Sciences–Geochemistry, Utrecht University, Utrecht, The Netherlands; 4 NIOZ Royal Netherlands Institute for Sea Research, Den Burg, The Netherlands

**Keywords:** Environmental impact, Environmental sciences, Ocean sciences, Marine biology

## Abstract

At present, the distribution of plastic debris in the ocean water column remains largely unknown. Such information, however, is required to assess the exposure of marine organisms to plastic pollution as well as to calculate the ocean plastic mass balance. Here, we provide water column profiles (0–300 m water depth) of plastic (0.05–5 cm in size) concentration and key planktonic species from the eastern North Atlantic Ocean. The amount of plastic decreases rapidly in the upper few meters, from ~ 1 item/m^3^ (~ 1000 µg/m^3^) at the sea surface to values of ~ 0.001–0.01 items/m^3^ (~ 0.1–10 µg/m^3^) at 300 m depth. Ratios of plastic to plankton varied between ~ 10^–5^ and 1 plastic particles per individual with highest ratios typically found in the surface waters. We further observed that pelagic ratios were generally higher in the water column below the subtropical gyre compared to those in more coastal ecosystems. Lastly, we show plastic to (non-gelatinous) plankton ratios could be as high as ~ 10^2^–10^7^ plastic particles per individual when considering reported concentrations of small microplastics < 100 μm. Plastic pollution in our oceans may therefore soon exceed estimated safe concentrations for many pelagic species.

## Introduction

Plastic debris accumulating in our oceans represents a pressing environmental issue. To date, plastic fragments have been found in virtually all marine ecosystems, yet the ecological risks of plastic pollution remain largely unknown^1^. To close this knowledge gap, a better understanding of the amount and types of plastics in the different oceanic compartments and the exposure of marine life is needed. After entering the ocean from land-based^2–6^ or maritime sources^7–11^, plastic debris is subjected to a wide range of physical and biological transport processes^12^. Plastic objects with a density higher than seawater sink toward the seabed, where they can subsequently be redistributed horizontally by, for example, deep-sea circulation^13^, turbidity currents^14^ and hyperpycnal flows^15^. The fate of positively buoyant plastic objects in the ocean, on the other hand, is largely dominated by beaching onto coastlines, which removes a large fraction of floating plastic from the ocean surface^16–22^. Initially buoyant plastic debris can further undergo changes in its buoyancy due to biofouling (i.e., the colonization with marine organisms)^23–28^ and weathering-induced chemical changes^29,30^. Thus, floating plastic objects that escape beaching can travel over large distances on the global scale, both horizontally^12,31–33^ and vertically within the ocean water column^34–37^.

The highest offshore concentrations of positively buoyant plastic debris have been recorded in the subtropical oceanic gyres^38–40^, where plastic concentrations can exceed hundreds of kilograms and a million pieces per km^2^ for particles > 500 µm in size. Trapped by large scale ocean circulation, floating plastic debris may persist in these subtropical surface waters for decades^16^, fragmenting into microplastics (< 5 mm) by the action of the sun, waves, temperature variations and marine organisms^29,41,42^. Some of these microplastics are subsequently lost to the underlying deep-sea through sedimentation^26,28,43–45^. How quickly and by which means these once buoyant microplastics are reaching deeper water layers and their residence time at specific water depths has not been fully resolved. Evidence of microplastics in the ocean water column indicates that pelagic organisms are exposed to plastic pollution at a range of depths^43,46–50^. However, the magnitude of the plastic abundance as well as organism’s exposure towards it and the potential ecotoxicological effects are still poorly understood.

Recent observations in the North Pacific Ocean revealed relatively higher plastic to organism ratios inside the North Pacific subtropical gyre for most members of the surface-associated pelagic community (hereafter collectively referred to as neuston,^51^) compared to waters outside the subtropical gyre^52^. These first findings indicate that neuston residing within subtropical oceanic gyres could be more likely to interact with floating plastic debris than organisms outside the gyres. The observations in the North Pacific Ocean further showed that the primary neustonic species likely to be found in higher concentrations with floating plastic in the subtropical gyre were those carried by the same forces as the plastic (i.e., currents) and those benefitting from the presence of these floating objects (e.g., for laying eggs or for habitat). To assess the extent to which the findings in the North Pacific Ocean can be generalized for other oceans, more observational data from other subtropical oceanic gyres are needed. Furthermore, little is known about plastic to organism ratios in the water column below subtropical gyres and how these ratios compare to the ones in more coastal pelagic ecosystems.

Here, we provide water column profiles (0–300 m water depth) of plastic debris (> 500 µm) and key planktonic species from the North Atlantic Ocean based on Manta trawl and multinet samples taken at twelve stations along a cruise transect from the North Atlantic subtropical gyre to the Netherlands. Our results reveal new insights into the vertical (mass and numerical) distribution and composition of plastic, as well as on specific plastic-to-organism ratios of planktonic species present in the North Atlantic water column.

## Methods

### Sampling

**Figure 1:**
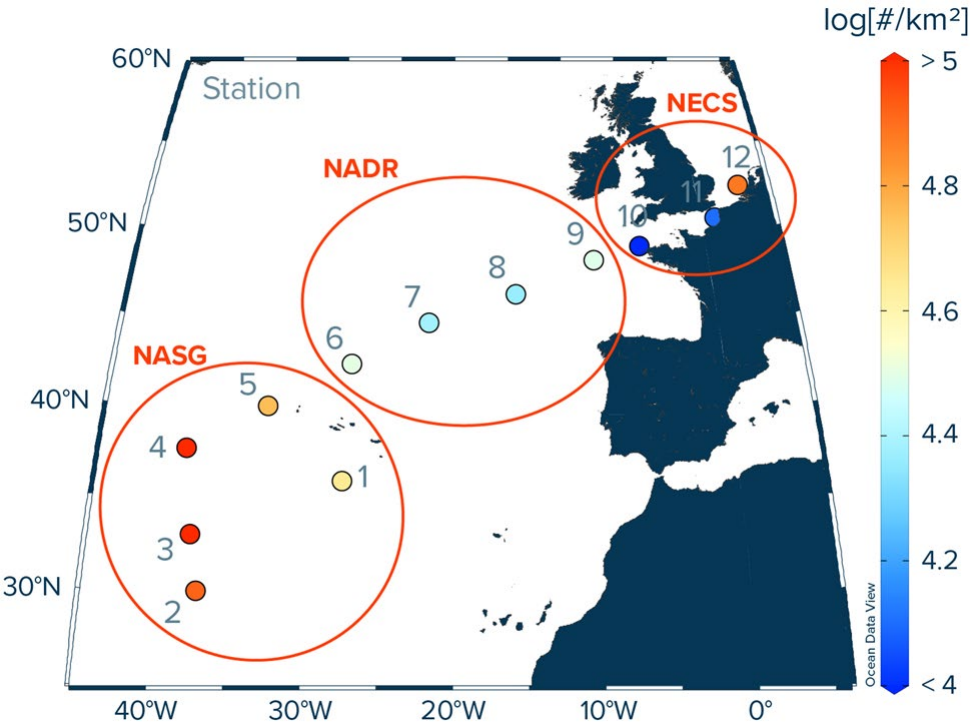
Locations of study sites in the eastern North Atlantic Ocean and associated measured numerical concentrations of floating plastic debris [#/km^2^] at the ocean surface (debris size: 500 µm to 5 cm in size). The numbers 1–12 correspond to the station numbers. Note that the numerical concentrations shown here represent average values of three Manta trawl deployments per station. All values were corrected for wind-induced mixing (see Supplementary Information for calculations). *NASG* North Pacific Subtropical Gyre, *NADR* North Atlantic Drift Region, *NECS* Northeast Atlantic Continental Shelves. The map was created using Ocean Data View (version 5.5.2; https://odv.awi.de/).

Vertical concentration profiles of plastic debris (> 500 µm) and plankton in the upper 300 m of water column were collected onboard RV Pelagia during the 64PE480 Expedition in November–December 2020. Samples were taken at twelve stations along a cruise transect from the Azores (Ponta Delgada, São Miguel) to the Netherlands (Texel) across the eastern portion of the North Atlantic subtropical gyre (Fig. 1). At each Station, a hydrocast with a CTD (Conductivity, Temperature, Depth) profiler (Sea-Bird SBE911 +) was conducted for measuring temperature, salinity, oxygen concentrations, and chlorophyll fluorescence. The water column profiles of these parameters were used to identify distinct water layers/masses of interest such as the mixed layer and the chlorophyll maximum.

The ocean surface was sampled with a Manta trawl (Ocean Instruments, Inc., Fall City, USA) deployed from the starboard crane (to avoid potential contamination from the vessel), sailing at around 1.5 knots. The Manta trawl mouth area was 90 cm × 15 cm (width × height), and the net mesh size was 500 µm (square). Three consecutive trawls, each 20 min in duration, were performed and towed distance was recorded using a mechanical flow meter (General Oceanics, Inc.). After each deployment, the net was rinsed from the outside with seawater and the cod-end (333 µm mesh size) was removed, sealed with staples, placed in a zip-lock bag, wrapped in aluminum foil, and stored frozen (− 18 °C) until further analysis in the onshore laboratory. The average trawling distance (± 1 standard deviation) for each Manta trawl deployment was 0.96 ± 0.12 km.

Samples from the ocean water column were taken by deploying a multinet (Hydrobios, Altenholz Germany) from the stern over the A-frame. The multinet consisted of a total of 5 individual nets with a mesh size of 200 µm (square). The net aperture dimensions were 50 cm × 50 cm. During each deployment, up to 5 water depths were sampled within the upper 300 m of the water column. Each water depth was sampled by towing for 20–30 min at around 1.5 knots. Depth for each net was maintained within 3 m of the target depth by monitoring the real-time data from a depth sensor mounted on the net frame and dynamically adjusting the length of the tow wire. At the end of each individual net tow at depth, the net was closed and the subsequent net opened remotely via a signal to the net frame. Upon retrieval on deck, each net was rinsed from the outside with seawater and the individual cod-ends (100 µm square mesh) were removed, their content transferred to Whirl-Paks, sealed, wrapped in aluminum foil, and stored frozen (− 18 °C) until further analysis in the onshore laboratory. All net tows were conducted during daylight hours in the afternoon.

To evaluate the relative distribution of small (0.05–5 cm) and large (> 50 cm) floating plastic debris (such as crates, ghost nets, or buoys), we mounted a GPS enabled camera (GoPro Hero 6 black) on the starboard side of the vessel bridge deck (platform height: 8.75 m, field of view: 24 mm focal length with 49.8 degrees horizontal FOV) collecting geo-tagged images of the ocean surface. The camera recorded photo time-lapses with intervals of 2 s. Back onshore, the photos were quantitatively analyzed for floating megaplastic debris (> 50 cm) by applying a previously developed object detection algorithm^53^.

### Sample processing

All samples were analyzed using the same analytical protocol as previously published^54^ to enable comparability with previous research in the eastern North Pacific Ocean. Briefly, each Manta trawl sample was thawed then washed into a sieve tower comprising four round stainless-steel sieves (diameter: 29 cm; mesh sizes: 15 mm, 5 mm, 1.5 mm and 0.5 mm, all square). The individual sieves were then placed into round aluminum tins (356 mm diameter, 76 mm height) filled with filtered seawater (< 1 µm) from the North Atlantic Ocean. Multiple LED lights were placed over the sieves from various angles to ensure good lighting conditions, which is particularly important for detecting small microplastics and organisms. Subsequently, all particles as well as all organisms were identified with the naked eye and hand-picked individually using stainless-steel tweezers.

The widest particle dimension was measured with a ruler and the particles were subsequently separated into the four size classes: (I) 0.05–0.15 cm, (II) 0.15–0.5 cm, (III) 0.5–1.5 cm, and (IV) 1.5–5 cm, respectively. Each particle was further classified and assigned to one of the following type categories: (1) ‘H-type’ for fragments and objects made of hard plastic, plastic sheet or film; (2) ‘N-type’ for fragments of plastic lines, ropes, and fishing nets; (3) ‘P-type’ for pre-production plastic pellets in the shape of a cylinder, disk or sphere; and (4) ‘F-type’ for fragments or objects made of expanded plastic. Once counted and categorized, the plastic objects were washed with water purified by reverse osmosis, transferred to aluminum dishes, dried at 65 °C for 3.45 h, and weighed using an OHAUS Explorer EX324M scale.

Organisms (typically varying between 0.05 and 5 cm in size) were further inspected under a light microscope (Leica DMC2900) and morphologically identified with the aid of in-house zooplankton guides^55,56^ and allocated to taxonomic groups as was done in^52^: *Velella velella*, *Halobates* spp., *Janthina janthina*, *Porpita porpita*, *Glaucus* spp., siphonophores, copepods, amphipods, pteropods, isopods, heteropods, crabs, squid, euphausiids and shrimps, and fish. In addition, the categories chaetognaths, and salps were added, and the occurrence of foraminifera, ostracods, fish eggs, and juvenile barnacles was noted (see Supplementary Information). Note that *Sargassum* was typically removed from the trawl samples onboard and is therefore not considered in this study.

The multinet samples were analyzed using the same procedures as outlined above for the Manta trawl samples. An additional 100 µm stainless-steel sieve was added to the sieve tower to account for the finer mesh size of the multinet (i.e., 200 µm) compared to the Manta trawl net (500 µm). To enable comparability between the multinet and Manta trawl samples, only particles > 500 µm were used for the subsequent analyses.

The particles extracted from the Manta trawl and multinet samples were analyzed using Raman spectroscopy (Agiltron, Inc., PeakSeeker PEK-785 and Thermo Scientific DXR3) to identify the corresponding plastic polymer types. While all particles from the multinet samples were analyzed, only a subset of particles was analyzed from the Manta trawl samples. For the latter, we analyzed a subset of 10 particles if the number of particles per size class and type category exceeded 10 pieces. In total, 92 and 199 particles were analyzed by Raman spectroscopy for the multinet and Manta trawl samples, respectively. Particles identified using the PeakSeeker Raman were compared to both in-house and published Raman polymer reference libraries^57^. Particles that could not initially be identified were analyzed once more by the ThermoFisher Raman microscope, and resulting spectra were scored using the OMNIC Spectra software against both in-house and provided polymer libraries (Raman Polymer Spectral Library, Thermo Scientific Catalog number: 834–014,101). For all spectra, a minimum match of 75% was used to positively identify the polymer.

The numerical and mass concentrations of plastic items measured by each Manta trawl net tow were corrected for wind-induced turbulent mixing^34^ (see Supporting Information (SI) for calculations). Furthermore, the detection limit was defined as a minimum of one particle collected by the trawl. Measures taken to minimize contamination during sampling and sample processing are described in the SI. We further performed a series of pre- and post-deployment blanks to evaluate potential plastic contamination or particle loss during sampling (Supplementary Table S1). Considering that most fragments > 500 µm are visible to the naked eye using good light conditions and that plastic microfibers were not part of the scope of our study, it is unlikely that a significant fraction of non-microfiber microplastics > 500 µm was missed using the methods applied here.

To calculate taxon specific plastic to organism ratios, we divided the number of plastic particles by the sum of individuals present in each tow sample for each group of organisms. Thus, these plastic to organism ratios are based on uncorrected plastic concentration values to allow for comparisons of equivalent measures (i.e., only comparing what was caught in the Manta trawl in particular conditions).

To evaluate observational patterns along our cruise transect, we grouped our sampling sites by the corresponding oceanographical province^58^. Stations 1–5 were assigned to the North Atlantic subtropical gyre (NASG), while stations 6–9 and 10–12 were assigned to the North Atlantic Drift Region (NADR) and the Northeast Atlantic Continental Shelves (NECS), respectively (Fig. 1).

## Results

### Plastic concentrations

In total, 679 plastic particles were collected from the ocean surface by Manta trawling. Measured numerical concentrations of plastic debris (0.05–5 cm in size) afloat at the ocean surface were highest in the NASG, with an average of 95,017 particles (#) per km^2^ (Fig. 1). However, within the NASG, the observed numerical abundances varied from values below detection limit to 333,606 #/km^2^. Surface waters in the NADR had the lowest average plastic concentration of 27,192 #/km^2^, with values ranging from below detection limit to 42,545 #/km^2^. Concentrations of floating plastic debris increased again in the waters of the NECS, with an average value of 39,485 #/km^2^ and an observed range of below detection limit to 97,572 #/km^2^. The mass concentrations of plastic debris (0.05–5 cm in size) afloat in the surface waters showed similar trends (Supplementary Fig. S1), with highest mass concentrations in the NASG (average: 552 g/km^2^, range: below detection limit to 2,937 g/km^2^), lowest in the NADR (average: 69 g/km^2^, range: below detection limit to 191 g/km^2^), and intermediate values in the NECS (average: 100 g/km^2^, range: below detection limit to 334 g/km^2^). The corresponding volumetric mass and numerical concentrations integrated over the upper 0–5 m of water column (i.e., the wind-mixed layer^34^) are shown in Table 1.

**Table 1:** Average numerical and mass concentrations of plastic debris (0.05–5 cm in size) observed in distinct water layers of three different biogeochemical provinces in the North Atlantic Ocean and corresponding plastic type and size distributions (in % of total plastic particle counts).

	Plastic concentration		Type category				Size category			
	*10^–3^ (# /m^3^) (min–max)	(µg/m^3^) (min–max)	H (%)	N (%)	P (%)	F (%)	0.05–0.15 cm (%)	0.15–0.5 cm (%)	0.5–1.5 cm (%)	1.5–5 cm (%)
**NASG**										
0–5 m	19.0 (< 0.4–66.7)	110.5 (< 5.3–587.4)	96	1	2	1	13	65	20	3
5–100 m	9.7 (< 2.5–19.7)	4.6 (0.8–9.6)	35	62	0	3	18	71	12	0
100–300 m	9.4 (2.6–24.5)	4.5 (0.4–27.3)	37	63	0	0	44	52	4	0
**NADR**										
0–5 m	5.4 (< 1.4–8.5)	13.8 (< 3.2–38.2)	88	11	1	0	19	63	15	2
5–100 m	3.5 (< 2.2–4.1)	0.5 (0.4–0.6)	50	50	0	0	50	50	0	0
100–300 m	9.1 (< 1.7–22.3)	6.5 (0.5–21.6)	35	65	0	0	41	41	6	12
**NECS**										
0–5 m	7.9 (< 0.9–19.5)	20.0 (< 0.3–66.9)	93	4	0	3	41	51	4	4
5–100 m	4.4 (< 2.1–8.1)	1.4 (0.2–2.2)	30	70	0	0	30	50	10	10

Values in parentheses refer to the minimum and maximum measured concentration in the respective water layer and province. NASG = North Atlantic Subtropical Gyre, NADR = North Atlantic Drift Region, NECS = Northeast Atlantic Continental Shelves. Plastic categories correspond to fragments and objects made of hard plastic (H), fragments of plastic lines, ropes, and fishing nets (N), pre-production plastic pellets (P), and fragments or objects made of foamed material (F).

**Figure 2:**
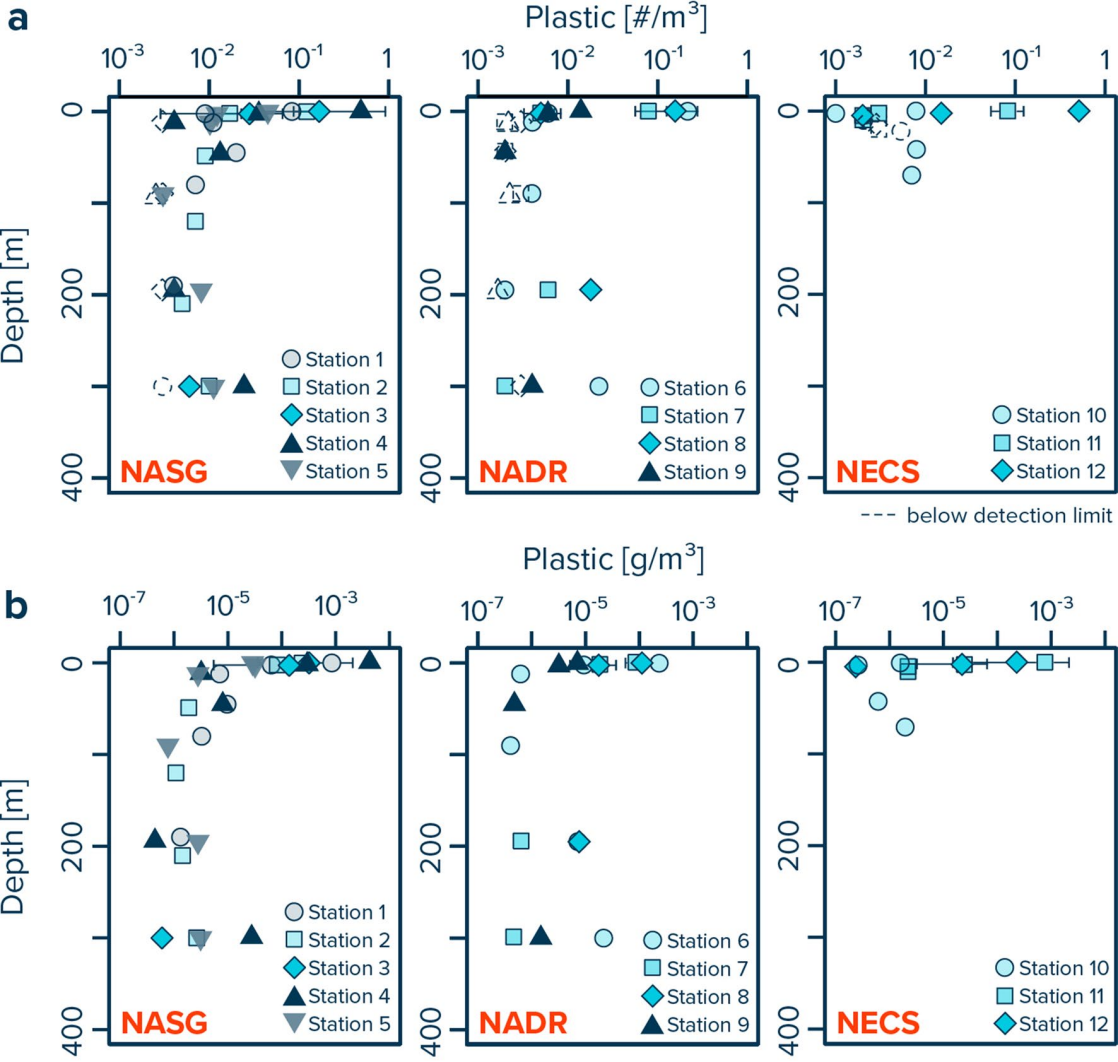
Water column profiles of (**a**) numerical and (**b**) mass concentrations of plastic particles between 0.05 and 5 cm in size. Data collected with Manta trawls are presented for the upper 0.15 m (net opening) of the ocean surface, and as values corrected for wind-induced mixing in the upper 5 m of water column, with average concentrations and whisker extending to the smallest and largest concentrations measured. Dashed symbols represent the detection limit for multinet samples in which no plastic fragments were found. *NASG* North Atlantic Subtropical Gyre, *NADR* North Atlantic Drift Region, *NECS* Northeast Atlantic Continental Shelves.

A total of 92 individual plastic particles (0.05–5 cm in size) were collected from the ocean water column (5–300 m depth in the NASG and NADR; 5–80 m depth in the NECS) by multinet underwater trawling across the twelve stations. At all sites, plastic concentrations decreased rapidly from ~ 1 #/m^3^ (~ 1,000 µg/m^3^) in the upper few meters of water column to between ~ 0.01–0.001 #/m^3^ (~ 0.1–10 µg/m^3^) at depth (Fig. 2). Lowest plastic concentrations were often found at water depths corresponding to the deep chlorophyl maximum (Supplementary Fig. S2). The observed mixed layer depth varied between ~ 70 and 80 m in the NASG and between ~ 70–100 m in the NADR (Supplementary Fig. S2). All samples taken from the NECS were within the mixed layer depth that reached to the seafloor (< 80 m water depth).

Fragments of hard plastics (i.e., H-type plastics) were the dominant debris type collected at the ocean surface, accounting for 96%, 88% and 93% of all floating plastic particles in the NASG, NADR and NECS, respectively (Table 1). In the water column, the contribution of N-type plastics (i.e., fragments of plastic lines, ropes and fishing nets) increased relative to the surface waters, accounting for between 50 and 70% of the collected water column plastic particles in the different regions, with the remaining particles mostly attributed to H-type plastics (Table 1).

With respect to particle size distribution, particles between 0.15 and 0.5 cm in size were the dominant size fraction in the surface waters, where they accounted for between 51–65% of floating particles across the three provinces (Table 1). The contribution of smaller particles (i.e., 0.05–0.15 cm) was between 13 and 19% in the surface waters of the NASG and NADR, and 41% in the surface waters of the NECS. The relative abundance of smaller particles generally increased with water depth in the NASG and NADR, accounting for > 40% in the deepest water layer (i.e., 100–300 m water depth).

**Figure 3:**
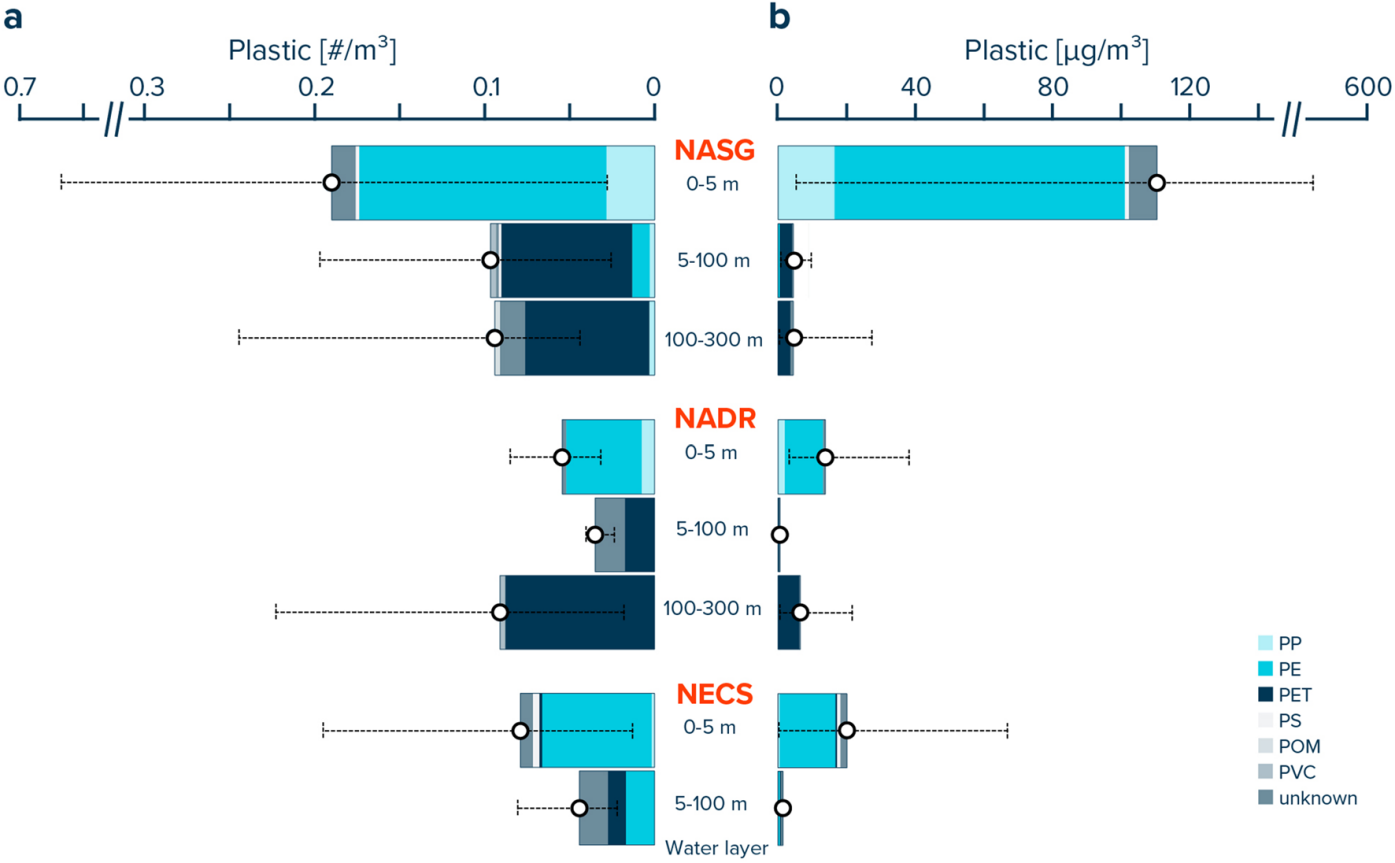
Vertical distribution of (**a**) numerical and (**b**) mass concentrations of plastic debris (0.05–5 cm in size) for specific water layers in the eastern North Atlantic Ocean and corresponding polymer composition. White dots represent average values and whisker extend to the smallest and largest concentrations measured in the respective depth layer. *NASG* North Atlantic Subtropical Gyre, *NADR* North Atlantic Drift Region, *NECS* Northeast Atlantic Continental Shelves.

The dominant plastic polymer type collected in the surface ocean was polyethylene (PE), accounting for 77–82% of all plastic particle (Table 2, Fig. 3). While polypropylene (PP) accounted for most of the remaining particles collected afloat in the NASG and NADR, surface waters in the NECS also contained polyethylene terephthalate (PET) and polystyrene (PS). Particles collected from the ocean water column were mostly made from PET. However, some PE and PP particles were also found in the water column, particularly at 5–100 m water depths in the NASG and NECS. No PE or PP particles were identified from the NADR water column.

**Table 2:** Polymer composition of plastic particles collected by Manta trawling (0–5 m) and multinet underwater trawling (5–300 m).

	# Particles	Polymer composition						
		PP (%)	PE (%)	PET (%)	PS (%)	POM (%)	PVC (%)	Unknown (%)
**NASG**								
0–5 m	104	15	77	0	1	0	0	7
5–100 m	34	3	11	80	1	0	4	1
100–300 m	27	3	0	78	0	3	0	16
**NADR**								
0–5 m	52	14	81	0	0	0	0	4
5–100 m	4	0	0	50	0	0	0	50
100–300 m	17	0	0	96	0	0	4	0
**NECS**								
0–5 m	43	2	82	2	5	0	0	9
5–100 m	10	0	38	24	0	0	0	38

Note that while all particles from the multinet samples were analyzed, a subset of particles was analyzed from the Manta trawl samples. For the latter, if the number of particles per size class and type category exceeded 10 pieces, a random subset of 10 particles was analyzed. Raman spectra for which no reference could be allocated due to low spectra quality were labelled as “unknown”. *NASG* North Atlantic Subtropical Gyre, *NADR* North Atlantic Drift Region, *NECS* Northeast Atlantic Continental Shelves.

Concentrations of floating megaplastic debris (i.e., > 50 cm) derived by analyzing the GoPro footage with the object-detection model developed by^53^ followed the observed patterns in micro- and mesoplastic debris afloat at the ocean surface, with highest concentrations in the NASG and lowest in the NADR (Supplementary Fig. S3). Average concentrations of floating megaplastics were 8.4 #/km^2^ (range: 0.2–36.0 #/km^2^) in the NASG, 0.2 #/km^2^(range: 0.1–0.2 #/km^2^) in the NADR, and 0.8 #/km^2^ (range: 0.4–1.0 #/km^2^) in the NECS. Although concentrations showed a high variability in the surface waters of each biogeographic region, both for smaller (< 5 cm) as well as for larger (> 50 cm) plastic debris, we observe a high correlation (R^2^ = 0.992) between average values of the two size classes (Supplementary Fig. S4).

### Relative distribution of plastic and pelagic organisms

The surface waters of the NECS, NADR and NASG showed distinct differences in the composition of neuston and corresponding numerical abundances of different members of neuston (Table 3). Species such as copepods, amphipods, isopods, euphasiids and shrimps, fish, and salps were present in Manta trawls across all three provinces. Other species such as *P. porpita*, heteropods, squid, and siphonophores were only observed inside the NASG. The presence of *V. velella* and pteropods was restricted to surface waters of the NADR and NASG, whereas crabs and chaetognaths were only observed in Manta trawls collected within the NECS and NASG. No species of *Halobates*, *J. janthina*, or *Glaucus* spp. were found in the trawl samples collected in this study.

**Table 3:** Median numerical abundance [individuals/km^2^] of different members of the neuston observed in surface waters of the Northeast Atlantic Continental Shelves (NECS), the North Atlantic Drift Region (NADR) and the North Atlantic Subtropical Gyre (NASG), respectively.

	NECS	NADR	NASG
V.velella	< LOD	2,632 *(1,652–6,956)*	7,014 *(4,290–15,657)*
P. porpita	< LOD	< LOD	2,501 *(2,093–4,209)*
Copepods	127,621 *(14,769–1,069,966)*	7,964 *(2,797–12,651)*	29,888 *(19,159–75,866)*
Amphipods	9,472 *(6,715–12,383)*	25,535 *(15,267–451,488)*	5,404 *(1,988–10,910)*
Pteropods	< LOD	4,537 *(2,345–4,728)*	4,119 *(1,848–7,543)*
Isopods	8,990 *(4,123–14,769)*	1,199 *(1,190–1,207)*	1,231 *(1,165–1,762)*
Heteropods	< LOD	< LOD	1,438 *(1,351–6,253)*
Crabs	2,927 *(2,178–5,358)*	< LOD	3,154 *(1,193–5,115)*
Squid	< LOD	< LOD	1,099 *(1,075–2,426)*
Euphasiids and shrimps	31,775 *(19,059–46,172)*	1,631 *(1,196–4,195)*	28,831 *(17,011–44,696)*
Fish	3,116 *(1,241–3,165)*	4,565 *(2,119–7,076)*	4,849 *(2,370–32,231)*
Chaetognaths	7,490 *(1,177–23,886)*	< LOD	2,785 *(1,851–4,327)*
Siphonophores	< LOD	< LOD	7,173 *(2,982–9,610)*
Salps	1,039*	31,035 *(16,541–45,529)*	1,351 *(1,138–2,472)*
Fish eggs	11,021 *(8,592–28,027)*	959*	1,231 *(1,184–1,433)*

*Based on one value only (i.e., species was only found in one out of n samples).

Values in parentheses refer to the 25th and 75th percentiles. *LOD* limit of detection (average: 1′174 individuals/km^2^ for all Manta trawl deployments, range: 959–1′504).

Many members of the neuston (i.e., *V. velella*, copepods, pteropods, isopods, euphasiids and shrimps) showed highest median plastic to organism ratios in the NADR compared to surface waters in the NECS and NASG (Supplementary Fig. S5). Other neuston such as amphipods, fish, and salps had highest median plastic to organism ratios in the NECS. Ratios of plastic to chaetognaths were highest in the NASG. No comparison of plastic to organism ratios between the three provinces was possible for *P. porpita*, heteropods, squid and siphonophores, as these species were only observed in the surface waters of the NASG.

**Figure 4:**
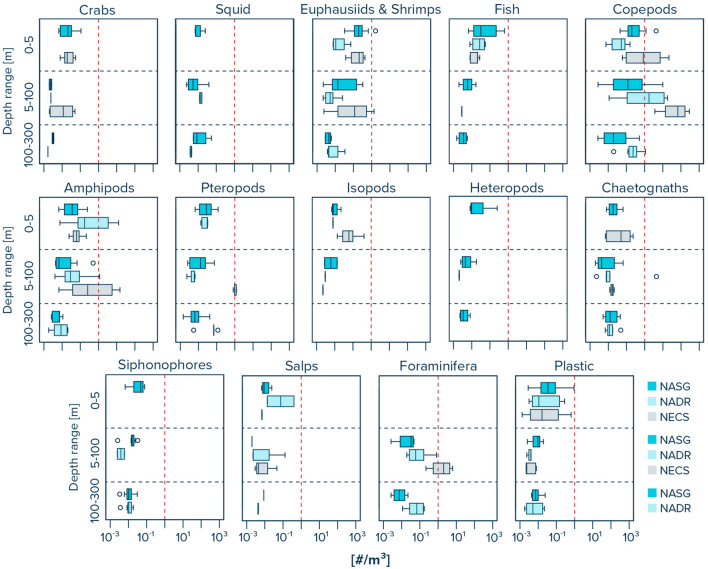
Observed water column distribution of marine organisms for three specific water layers (i.e., 0–5 m, 5–100 m and 100–300 m) in the North Atlantic Subtropical Gyre (NASG), the North Atlantic Drift Region (NADR), and the Northeast Atlantic Continental Shelves (NECS). Solid vertical lines represent median values. Box plots extend from the 25th to the 75th percentiles, while whiskers extend from the minimum to the maximum observed values. Dots indicate outliers. Red dashed vertical line represents x-axis value of 1 for better visual comparison between the taxa. Note that all values are provided in Supplementary Table S2.

**Figure 5:**
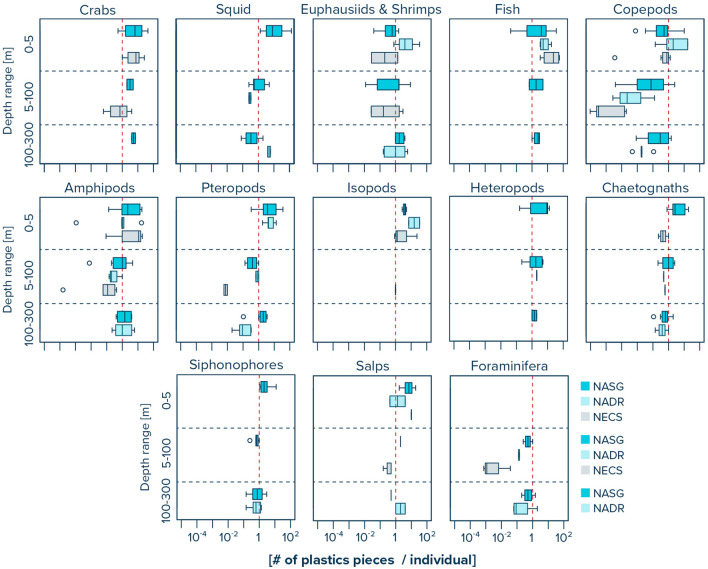
Observed water column distribution of plastic to organism ratios for three specific water layers (i.e., 0–5 m, 5–100 m and 100–300 m) in the North Atlantic Subtropical Gyre (NASG), the North Atlantic Drift Region (NADR), and the Northeast Atlantic Continental Shelves (NECS). Solid vertical lines represent median values. Box plots extend from the 25th to the 75th percentiles, while whiskers extend from the minimum to the maximum observed values. Dots indicate outliers. Red dashed vertical line represents x-axis value of 1 for better visual comparison between the taxa. Note that all values are provided in Supplementary Table S3.

The abundance distribution of pelagic organisms in the ocean water column was different between distinct water layers (Fig. 4, Supplementary Table S2). Species such as crabs, fish, isopods, heteropods, siphonophores, and salps generally showed highest abundances in the upper 0–5 m of the water column across all three provinces. In contrast, foraminifera were only observed in the multinet samples, i.e., at water depths below 5 m. We note that this is likely due to the finer net mesh size of the multinet underwater trawl (200 µm) compared to the Manta trawl (500 µm). Squid were only observed in the water column of the NASG and the NADR, with slightly higher abundances at depths between 100–300 m in the NASG and at depths of 5–100 m in the NADR, respectively. The abundance of euphasiids and shrimps generally decreased with tow depth in all three provinces. Copepods decreased in abundance with increasing tow depth in the NASG, while their abundance was highest at tow depths between 5 and 100 m in the NADR and NECS. Amphipod abundance decreased with increasing tow depth in the NASG and NADR, but was highest at tow depths of 5–100 m in the NECS. The abundance of pteropods was highest in the upper 0–5 m in the NASG, at 5–100 m in the NECS, and at 100–300 m in the NADR, respectively. Chaetognaths were lowest in abundance at 5–100 m tow depth in the NASG and NECS, and at 0–5 m tow depth in the NADR. Plastic to organism ratios within the water column were typically highest in the surface waters (0–5 m depth) and lowest at depths of 5–100 m in all three provinces (Fig. 5, Supplementary Table S3).

## Discussion

### Floating plastic debris

The spatial distribution of floating plastic debris observed in our study is in good agreement with earlier predictions of plastic pollution in the surface waters of the North Atlantic Ocean^38,40^. As expected, highest surface concentrations were found in the NASG. Numerical concentrations of floating microplastics measured in this study are on average around 4 times higher than previously predicted by global models^38^, with the exception of Stations 10 and 11 in the NECS that were on average 17% lower than predicted (Supplementary Fig. S6). Nevertheless, our measured microplastic (particle size range: 500 µm–5 mm) mass concentrations typically fall within an order of magnitude of values predicted by global models (particle size range: 330 µm–4.75 mm; Supplementary Fig. S6). The agreement between measured and predicted microplastic mass concentrations was highest in the NADR and lowest at Stations 10 and 11 located in the NECS. Considering that concentrations of floating plastic debris were highly variable between consecutive Manta trawl deployments (which were on average only ~ 1 km apart) particularly for surface waters in the NASG (Supplementary Fig. S7), we consider the match between our measured mass concentrations and those predicted earlier by global plastic dispersal modelling as fair.

Our findings of variable plastic concentrations in Manta trawl samples, together with the high variability of megaplastic densities as observed here (Supplementary Fig. S4), strengthen previous indications that sub-mesoscale accumulation of floating plastic debris at the ocean surface is highly variable^43,59–61^. Such high spatial heterogeneity of plastic density at the ocean surface highlights the need to evaluate the influence of sub-mesoscale variability on global quantification estimates of floating plastic debris in the ocean. At present, such assessments are largely based on data from single surface net tows and visual surveys taken tens to hundreds of kms and often several years apart^38–40^. We therefore advocate for a more systematic assessment of the heterogeneity of plastic debris accumulation at the ocean surface. Neuston trawls should, whenever possible, be deployed in a series of at least three subsequent deployments to account for part of the high spatial variability of floating plastic densities on a sub-mesoscale. Our findings further support the use of vessel-mounted cameras to efficiently survey large ocean surface areas for larger floating plastic debris^53^.

### Water column plastic debris

**Figure 6:**
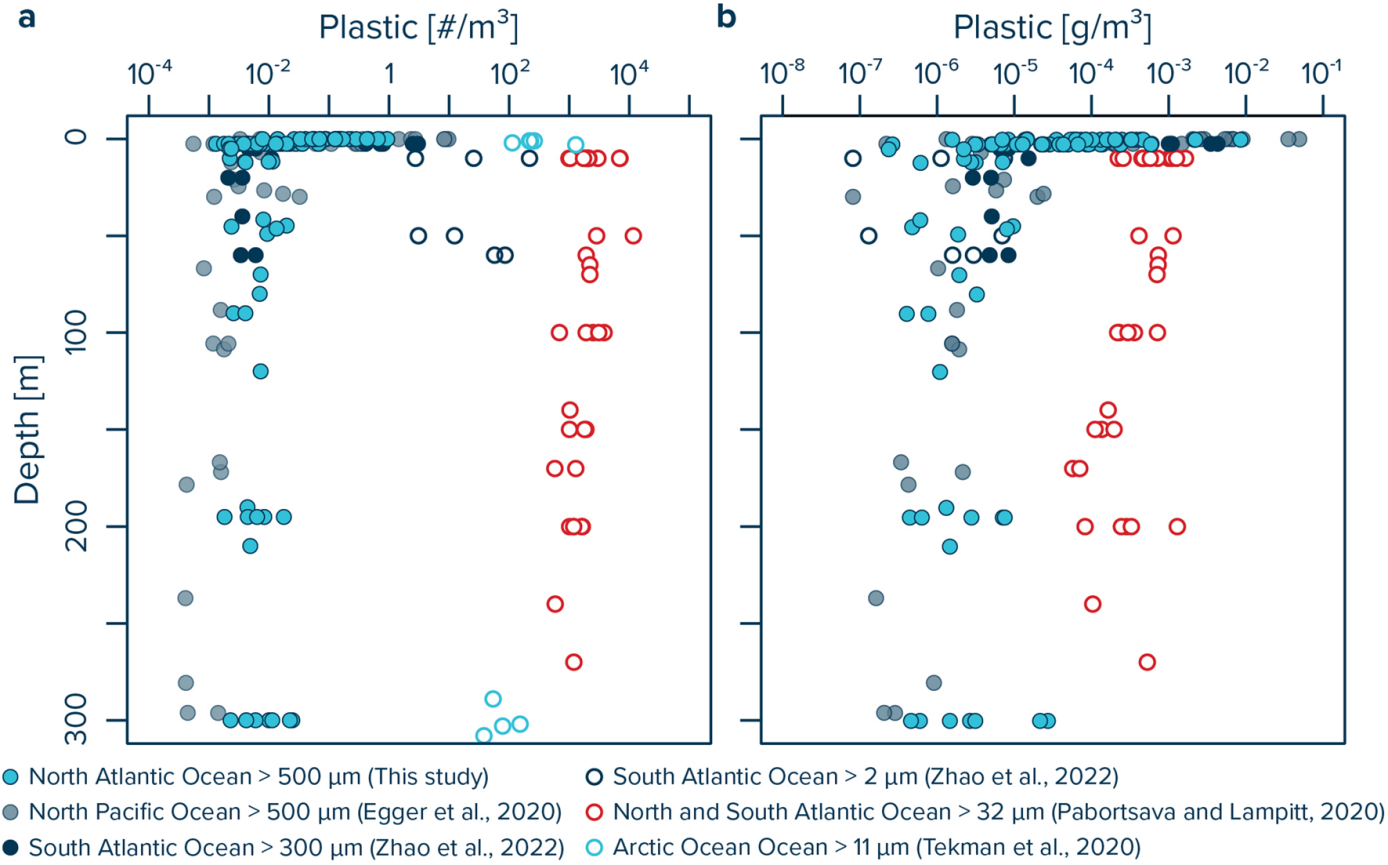
Comparison of (**a**) numerical and (**b**) mass concentrations of plastic debris of different sizes measured in the upper 300 m of ocean water column in this and other studies. Numerical concentrations of larger microplastics (> 100 µm) collected by underwater trawls^43,50^ can be up to 7 orders of magnitude lower than numerical concentrations of small microplastics (< 100 µm) collected by in-situ pumps^48–50^. Mass concentrations of small microplastics (< 100 µm) show a large range, varying by up to 5 orders of magnitude between two studies in the Atlantic Ocean^48,50^.

Pelagic plastic concentrations observed in this study vary between ~ 0.01 and 0.001 #/m^3^ at depths > 5 m, corresponding to ~ 0.1–10 µg/m^3^. We further observe lower plastic concentrations below the mixed layer down to 200 m in the NASG and NADR water column (Supplementary Fig. S2). This could be due to a number of reasons, including possible biological removal (e.g. uptake) of plastic particles from these water layers. Our values are within the same range as concentrations reported for plastic particles of similar sizes in the upper 300 m of the South Atlantic subtropical gyre (particles > 300 µm,^50^) and of the North Pacific subtropical gyre (particles > 500 µm,^43^) (Fig. 6). It is important to note, that such a comparison is only meaningful if the corresponding lower particle size limit is taken into account due to a general increase in abundance of microplastics with decreasing particle size^40,62,63^. Indeed, reported concentrations of small microplastics < 100 μm in the upper 300 m of the Atlantic and Arctic Oceans are much higher than concentrations of microplastics > 500 μm, with values ranging from tens to thousands of microplastic particles per m^3^^48–50,63^ (Fig. 6). As recently shown in the South Atlantic Ocean by Zhao and co-workers^50^, abundances of small microplastics < 100 μm in pump samples can be more than two orders of magnitude higher than larger microplastics > 300 μm concurrently collected in multinet samples. The reported water column plastic mass concentrations by these authors are generally at the lower range of mass concentrations reported for larger microplastics > 500 μm in the North Pacific and Atlantic Oceans (Fig. 6). Thus, the findings of ^50^ indicate that although small microplastics depict much higher numerical abundances, their mass concentrations are equal or less than those of larger microplastics. In contrast, Pabortsava and Lampitt^48^ report mass concentrations for small microplastics < 100 μm that are two to three orders of magnitude higher than observed mass concentrations for larger microplastics, thus suggesting high mass loads of microplastics in the ocean interior. These two contrasting findings highlight the need for more observational data on the mass contribution of microplastics in the ocean water column, particularly for microplastics < 100 μm.

### Plastic characteristics

The dominance of PE and PP as the main plastic polymers found at the ocean surface in this study is in line with current literature on plastic debris afloat in offshore waters^64^. We further observe a high contribution of PET particles in the water column, particularly in the NASG and NADR. Such a dominance of PET particles in the water column has also previously been reported in the Monterey Bay pelagic ecosystem for microplastics > 100 μm^47^. It is, however, in contrast to the observations in the North Pacific subtropical gyre, where plastic particles > 500 μm found in the water column were dominated by PE and PP^43^. The absence of detectable PET in our samples collected at the ocean surface in the NASG and NADR (Table 2, Fig. 3) could point towards a lateral source of PET particles at depths below 5 m. We hypothesize that due to their high density, PET particles quickly sink below the sea surface when entering the ocean and that they are subsequently transported offshore horizontally at depth. The absence of PET particles in the water column below the North Pacific subtropical gyre can thus be explained by differences in the distance to, and/or magnitude of, PET emission sources between the subtropical gyres in the North Atlantic and North Pacific Oceans. It could, however, also derive from differences in the mesh size used to collect water column particles in the North Pacific subtropical gyre (333 µm,^43^) compared to net mesh sizes used in the Monterey Bay (100 µm,^47^) and in this study (200 µm). Water column particles identified as PET in this study typically were classified as N-type plastics. Given their fibrous rather than spherical shape, N-type plastics are likely more impacted by differences in the mesh size used to collect plastic particles from the ocean water column^62^. This could also, at least partly, explain the generally higher contribution of N-type plastics in the water column compared to the surface waters, both in the North Pacific^43^ and North Atlantic Oceans (Table 1). In both studies, the underwater trawls had finer mesh sizes (333 µm and 200 µm, respectively) compared to the Manta trawls (500 µm). Such a sampling bias associated with finer mesh sizes in the water column compared to the ocean surface could further explain our observed decrease in the average mass of plastic particles collected by Manta trawling vs multinet sampling (Supplementary Table S4). Due to their smaller volume, fibrous N-type plastics typically have a lower mass per particle compared to more spherical H-type plastics.

Removing all PET particles from the water column data set reveals that the numerical concentrations of plastic particles > 500 µm suspended in the upper 300 m of the North Atlantic Ocean generally follow a similar power law decline with water depth as observed for plastic particles > 500 µm in the North Pacific Ocean (Supplementary Fig. S8). Compared to the North Pacific Ocean, our measured plastic concentrations in the North Atlantic Ocean are lower at the ocean surface and higher in the water column. The enumeration of latter finding could be skewed due to the finer mesh size of the underwater trawl used in this study (200 µm) compared to the study in the North Pacific Ocean (333 µm). Alternatively, it could also indicate a more efficient transfer of microplastic particles from surface waters to the ocean interior at our study sites in the North Atlantic compared to the study sites in the North Pacific. While global plastic dispersal models show some vague support for such a difference in microplastic export efficiency^45^, more research is needed to evaluate relative microplastic export efficiencies from the surface in the North Pacific and North Atlantic Oceans.

### Neuston and floating plastic debris

The neustonic community composition observed in our samples shows some similarities to distribution patterns observed in the eastern North Pacific Ocean^52^. *P. porpita* and heteropods are restricted to surface waters in the subtropical gyre, while species such as copepods, amphipods, fish, euphasiids and shrimps were found at the ocean surface in all three regions (Table 3). Crabs are only observed inside the subtropical gyre and in more coastal waters. In contrast to the observations in the North Pacific Ocean, where neustonic isopods were only present inside the subtropical gyre, they were present in Manta trawl samples collected across all three North Atlantic provinces considered in this study. Furthermore, we also observe highest abundances of *V. velella* in the NASG (i.e., stations 1, 4, and 5), while they showed lowest abundances inside the North Pacific subtropical gyre. While our results highlight some differences in the spatial distribution for some species of the neuston between the eastern North Atlantic and North Pacific Oceans, and thus the need for more research on the life cycle dynamics of individual species of the neuston^51^, they do generally support the hypotheses by Egger and colleagues^52^ that passively drifting species with a low atmospheric drag (i.e., little protrusion above the sea surface) are more likely to co-occur with high concentrations of floating plastic debris in oceanic subtropical gyres due to a similar oceanic transport. Species with a higher vertical mobility, on the other hand, are likely to be found in surface waters both with low and high concentrations of floating plastic debris, as they migrate in search for nutrients and to avoid predation.

The findings reported here further reveal that neuston in the NASG coincides with lower plastic to organism ratios compared to neuston residing in the eastern North Pacific subtropical gyre (Supplementary Fig. S5). A higher exposure to plastic pollution indicates that neuston in the North Pacific subtropical gyre could be more likely to interact with floating plastic debris than in the NASG.

### Pelagic organisms and plastic

Plastic to organism ratios within the water column were typically highest in the surface waters (0–5 m depth) in all three provinces. We note, however, that many taxa are exposed to similar levels of plastic pollution throughout the upper 300 m in the open ocean (Fig. 5). Crabs, euphasiids and shrimps, fish, copepods, amphipods, pteropods, heteropods, chaetognaths, siphonophores, and foraminifera all show similar order of magnitude plastic to organism ratios in the upper 300 m of the NASG. We also find that copepods show 10–100 times higher plastic to organism ratios below the surface layer (i.e., < 5 m) in the NASG compared to the NADR, and up to 10,000 times higher ratios when compared to the NECS (Supplementary Table S3). This indicates a possible higher exposure of copepods to plastic pollution in the water column of the subtropical gyre compared to more coastal waters.

The ratio of plastic between 0.05 and 5 cm in size to organisms found in our study typically varied between ~ 10^–3^ and 10 plastic particles per individual for most taxa (Supplementary Table S3). It is important to note, however, that many taxa of zooplankton migrate vertically to deeper depths during daylight hours, and all of our samples were taken during the day. Consequently plastic to zooplankton ratios would be lower during the night. Nevertheless, recent studies focusing on small microplastics < 100 μm in the Atlantic and Arctic water column^48–50^ indicate that concentrations of small microplastics can be around 5–6 orders of magnitude higher than the number of microplastic particles > 500 μm measured in this study (Fig. 6). Consequently, the ratio of total plastic to meso- and macroplankton (500 μm to 5 cm) could be as high as ~ 10^2^–10^7^ plastic particles < 100 μm per individual. Integrating twenty-three species-specific effect threshold concentration data in a species sensitivity distribution, Everaert and co-workers^65^ calculated a median unacceptable level of ~ 10^5^ microplastic particles per m^3^ of seawater. Although observations are scarce, current data available shows that concentrations of small microplastics (< 500 μm) in the upper ocean (0–300 m depth) can vary between 10^1^ and 10^4^ #/m^3^^46,48–50^. With increasing accumulation of secondary microplastics in the global ocean^16^, microplastic pollution in our oceans may therefore soon exceed identified safe concentrations for pelagic life^65^, especially in sub-mesoscale plastic accumulation hotspots.

## Supplementary Information

Supplementary Information 1.

Supplementary Information 2.

## Data Availability

All data needed to evaluate the conclusions in the paper are present in the paper and/or the Supplementary Materials.
