# Supplementary material for: Pelagic distribution of plastic debris (> 500 µm) and marine organisms in the upper layer of the North Atlantic Ocean
Source: Sci Rep. 2022 Aug 11;12:13465. doi: 10.1038/s41598-022-17742-7 (PMC9372048; doi:10.1038/s41598-022-17742-7)
Supplement: Supplementary file 2 — Supplementary Information 2. [file 41598_2022_17742_MOESM2_ESM.pdf]

SUPPLEMENTARY INFORMATION

Pelagic distribution of plastic debris (> 500 µm) and marine organisms in the upper layer of the North Atlantic Ocean

Matthias Egger[1,2*], Britte Schilt[1,3], Helen Wolter[1], Thomas Mani[1], Robin de Vries[1], Erik Zettler[4], and Helge Niemann[3,4]

[1] The Ocean Cleanup, Rotterdam, The Netherlands

[2] Egger Research and Consulting, St. Gallen, Switzerland

[3] Department of Earth Sciences – Geochemistry, Utrecht University, Utrecht, The Netherlands

[4] NIOZ Royal Netherlands Institute for Sea Research, Den Burg, The Netherlands

*** Correspondence:**

Corresponding Author

matthias.egger@theoceancleanup.com

Keywords: Marine debris, ocean plastic pollution, microplastic, North Atlantic Ocean

Correction for Wind-induced Mixing

The numerical and mass concentrations of plastic items measured by each Manta net tow were calculated for each plastic size and type category separately. Positively buoyant plastic items are mixed within the upper water column due to wind-induced turbulent mixing [1–3]. As a result, plastic items collected by Manta trawling may underestimate the total amount of plastics afloat in the area sampled, especially at higher sea states. Kukulka and co-workers [1] developed a one-dimensional model that predicts the vertical distribution of buoyant plastic particles at different sea states. Their model can be applied to calculate depth-integrated numerical concentrations for the Manta trawl measurements in the upper 5 m of the water column, thus accounting for wind-driven mixing of buoyant plastics at the sea surface using the following equation [1]:

$$C_i = \frac{C_s}{1 - e^{-dW_b\left(1.5\sqrt{\frac{\rho_a}{\rho_w}C_d U^2}\, k \frac{0.96}{g} \sigma^{\frac{3}{2}} C_d U^2\right)^{-1}}} \qquad \text{(eq. 1)}$$

Where C_s represents the concentration of a plastic type and size category as measured by the Manta trawl (in pieces per surface area), d is the depth sampled by the Manta trawl (equal to 0.15 m), W_b is the terminal rising velocity of plastic within a plastic type and size category (in m/s) taken from [4], ρ_a is the air density (1.225 kg/m^3), ρ_w is the seawater density (1,024 kg/m^3), C_d is the drag coefficient (equal to 0.0012), U is the wind speed during sampling (in m/s), k is the Karman constant (equal to 0.4), g is the gravitational constant (equal to 9.81 m/s^2), and σ is the wave age, equal to 35 (assuming a fully developed sea state). Values for depth-integrated concentrations were estimated using wind speeds measured onboard, and the median values for rising velocity measurements provided in [4].

Contamination Controls

To minimize contamination with plastic fragments, standard non-plastic laboratory and fieldwork equipment such as metal and glass were used whenever possible, and the samples always remained covered with aluminum foil when not in use. Furthermore, all nets and all cod-ends were thoroughly rinsed from the outside prior to each deployment and carefully inspected for the presence of (micro)plastic particles. The Manta trawl was stored rolled up and closed between uses. The seawater (taken from the North Atlantic Ocean) used in the laboratory was filtered through a sequence of filters (< 20 µm, < 5 µm, < 1 µm) and all laboratory equipment (sieves, tweezers, aluminum tins and dishes) was thoroughly rinsed and carefully inspected for cleanliness prior to each use. Contamination by fibers broken off the nylon nets can be excluded as we did not observe any nylon fibers in the cod-end samples. Furthermore, we did not include any microfibers in our analyses, as these fibers typically pass through the trawling nets deployed here. Thus, possible contamination with airborne synthetic fibers (a major source of microplastic contamination) does not impact our results.

To evaluate potential plastic contamination during sampling with the Manta trawl, we performed a total of 2 pre- and 9 post-deployment blanks. Pre-deployment blanks were done by trawling the Manta trawl for 4-6 minutes without a cod-end, then attaching a fresh cod-end to the net and hosing it down from the outside. Post-deployment blanks were done by attaching a new cod-end to a Manta trawl that had just been sampled and by subsequently rinsing the net down again into the new cod-end. Seven pre-deployment blanks were done with the multinets by rinsing the net from the outside into a new cod-end for approximately 40 seconds. The results from these blank samples are summarized in Supplementary Table S1. One Manta trawl post-deployment blank contained a PE fragment in the 0.15 – 0.5 cm size fraction. No plastic fragments were found in the other blanks. However, we frequently observed blue paint chips in the blanks. Based on color and abundance, the majority of these paint chips likely originated from the vessel. Paint chips were not included in the plastic count. Three Manta

trawl blanks did contain copepods (< 5 individuals) and two contained amphipods (< 2 individuals). In the multinet samples, a blank control of the third multinet at station 7 revealed the presence of small (0.05 - 0.15 cm) orange filament-like fragments. Identical fragments were also observed in the third multinet at stations 6, 7, 9 and 10 (at station 8, the third multinet was lost due to a loose cod-end). The fragments possibly originate from the mounting ring of the multinet, therefore they were not included in the plastic count. Similar to the Manta trawls, some multinet blanks did contain paint chips. In addition, all multinet blanks contained small copepods. One multinet blank (Station 06, multinet 5/5) contained exceptionally high numbers of copepods (> 1,000), however the sample itself was lost due to a ripped cod-end. This is likely caused by the very high abundance of copepods at this location, causing clogging of the net.

Supplementary Table S1 | Results of Manta trawl and multinet blank samples.

	0.05 – 0.15 cm				0.15 – 0.5 cm				Total	Paint chips
	**H**	**N**	**P**	**F**	**H**	**N**	**P**	**F**	**[#]**	**[#]**
**Manta trawl**										
Station 6 pre-deployment blank	0	0	0	0	0	0	0	0	**0**	**1**
Station 6 post-deployment blank	0	0	0	0	0	0	0	0	**0**	**0**
Station 7 pre-deployment blank	0	0	0	0	0	0	0	0	**0**	**2**
Station 7 post-deployment blank	0	0	0	0	1	0	0	0	**1**	**1**
Station 8 pre-deployment blank	0	0	0	0	0	0	0	0	**0**	**3**
Station 8 post-deployment blank	0	0	0	0	0	0	0	0	**0**	**2**
Station 10 one-side rinse post-deployment trawl 3/3	0	0	0	0	0	0	0	0	**0**	**4**
Station 10 two-side rinse post-deployment trawl 3/3	0	0	0	0	0	0	0	0	**0**	**1**
Station 11 one-side rinse post-deployment blank	0	0	0	0	0	0	0	0	**0**	**0**
Station 11 two-side rinse post-deployment blank	0	0	0	0	0	0	0	0	**0**	**1**
Station 12 one-side rinse post-deployment trawl 3/3	0	0	0	0	0	0	0	0	**0**	**0**
Station 12 two-side rinse post-deployment trawl 3/3	0	0	0	0	0	0	0	0	**0**	**0**
**Multinet**										
Station 6 Multinet 2/5 pre-deployment blank	0	1	0	0	0	0	0	0	**1**	**0**
Station 6 Multinet 5/5 pre-deployment blank	0	0	0	0	0	0	0	0	**0**	**4**
Station 7 Multinet 1/5 pre-deployment blank	0	0	0	0	0	0	0	0	**0**	**0**
Station 7 Multinet 3/5 pre-deployment blank	0	0	0	0	0	4	0	0	**4**	**0**
Station 8 Multinet 5/5 pre-deployment blank	0	0	0	0	0	0	0	0	**0**	**0**
Station 8 Multinet 4/5 pre-deployment blank	0	0	0	0	0	0	0	0	**0**	**0**

Supplementary Table S2 | Median numerical taxon abundances [individuals/m^3] observed in different water layers of the Northeast Atlantic Continental Shelves (NECS), the North Atlantic Drift Region (NADR) and the North Atlantic Subtropical Gyre (NASG), respectively. Values in parentheses refer to the 25th and 75th percentiles. The upper 5 m of water column represent Manta trawl data, while waters > 5 m were sampled by multinet trawling. LOD = limit of detection, which was 0.008 (0.006 – 0.010) individuals/m^3 (average, min - max) for the Manta trawls and 0.003 (0.001 – 0.005) individuals/m^3 for the multinet, respectively.

	**NECS**		**NADR**			**NASG**		
**Species**	**0-5 m**	**5-100 m**	**0-5 m**	**5-100 m**	**100-300 m**	**0-5 m**	**5-100 m**	**100-300 m**
V. velella	< LOD	< LOD	0.018 *(0.011 – 0.046)*	< LOD	< LOD	0.047 *(0.029 – 0.10)*	< LOD	< LOD
P. porpita	< LOD	< LOD	< LOD	< LOD	< LOD	0.017 *(0.014 – 0.028)*	< LOD	< LOD
Crabs	0.020 *(0.015 – 0.036)*	0.017 *(0.003 – 0.036)*	< LOD	0.002*	< LOD	0.021 *(0.008 – 0.034)*	0.002 *(0.002 – 0.003)*	0.003 *(0.003 – 0.003)*
Squid	< LOD	< LOD	< LOD	0.014 *(0.013 – 0.015)*	0.004 *(0.004 – 0.004)*	0.007 *(0.007 – 0.016)*	0.005 *(0.003 – 0.009)*	0.009 *(0.006 – 0.024)*
Euphausiids & Shrimp	0.21 *(0.13 – 0.31)*	0.12 *(0.041 – 0.61)*	0.011 *(0.008 – 0.028)*	0.005 *(0.003 – 0.007)*	0.004 *(0.004 – 0.014)*	0.19 *(0.11 – 0.30)*	0.016 *(0.007 – 0.12)*	0.004 *(0.003 – 0.005)*
Fish	0.021 *(0.008 – 0.021)*	0.003*	0.030 *(0.014 – 0.047)*	< LOD	< LOD	0.032 *(0.016 – 0.21)*	0.006 *(0.004 – 0.011)*	0.004 *(0.002 – 0.005)*
Copepods	0.85 *(0.098 – 7.1)*	67 *(22 – 146)*	0.053 *(0.019 – 0.084)*	1.8 *(0.16 – 11)*	0.23 *(0.14 – 0.37)*	0.20 *(0.13 – 0.51)*	0.12 *(0.020 – 0.73)*	0.020 *(0.006 – 0.089)*
Amphipods	0.063 *(0.045 – 0.083)*	0.26 *(0.046 – 4.6)*	0.17 *(0.10 – 3.0)*	0.028 *(0.015 – 0.091)*	0.011 *(0.004 – 0.018)*	0.036 *(0.013 – 0.073)*	0.007 *(0.005 – 0.030)*	0.007 *(0.003 – 0.007)*
Pteropods	< LOD	1.1 *(0.98 – 1.2)*	0.030 *(0.016 – 0.032)*	0.006 *(0.004 – 0.006)*	0.070 *(0.069 – 0.071)*	0.027 *(0.012 – 0.050)*	0.013 *(0.006 – 0.022)*	0.006 *(0.005 – 0.009)*
Isopods	0.060 *(0.027 – 0.098)*	0.002*	0.008 *(0.008 – 0.008)*	0.003*	< LOD	0.008 *(0.008 – 0.012)*	0.008 *(0.005 – 0.011)*	< LOD
Heteropods	< LOD	< LOD	< LOD	0.002*	< LOD	0.010 *(0.009 – 0.042)*	0.004 *(0.003 – 0.008)*	0.003 *(0.003 – 0.005)*
Chaetognaths	0.050 *(0.008 – 0.16)*	0.016 *(0.014 – 0.019)*	< LOD	0.008 *(0.008 – 0.012)*	0.012 *(0.010 -0.017)*	0.019 *(0.012 – 0.029)*	0.004 *(0.003 – 0.020)*	0.013 *(0.008 – 0.026)*
Siphonophores	< LOD	< LOD	< LOD	< LOD	0.6 *(0.4 – 1.1)*	0.048 *(0.020 – 0.064)*	0.016 *(0.015 – 0.021)*	0.010 *(0.008 – 0.015)*
Salps	0.007*	0.004 *(0.004 – 0.015)*	0.21 *(0.11 – 0.30)*	0.002 *(0.002 – 0.066)*	0.004 *(0.004 – 0.004)*	0.009 *(0.008 – 0.016)*	0.002*	0.009*
Foraminifera	< LOD	2.2 *(1.1 – 3.9)*	< LOD	0.059 *(0.027 – 0.25)*	0.067 *(0.027 – 0.15)*	< LOD	0.033 *(0.021 – 0.042)*	0.008 *(0.005 – 0.013)*
**All**	**1.00** *(0.41 – 7.41)*	**70** *(25 – 154)*	**0.21** *(0.075 – 0.89)*	**1.8** *(0.41 – 13)*	**0.39** *(0.19 – 0.44)*	**0.69** *(0.52 – 1.7)*	**0.16** *(0.061 – 0.87)*	**0.065** *(0.037 – 0.14)*

* Based on one value only (i.e., species was only found in one sample in that region/depth)

Supplementary Table S3 | Median plastic to organism ratios [# plastic pieces per individual] observed in different water layers of the Northeast Atlantic Continental Shelves (NECS), the North Atlantic Drift Region (NADR) and the North Atlantic Subtropical Gyre (NASG), respectively. Values in parentheses refer to the 25th and 75th percentiles. The upper 5 m of water column represent Manta trawl data, while waters > 5 m were sampled by multinet trawling.

	**NECS**		**NADR**			**NASG**		
**Species**	**0-5 m**	**5-100 m**	**0-5 m**	**5-100 m**	**100-300 m**	**0-5 m**	**5-100 m**	**100-300 m**
V. velella	-	-	8.5 *(1.0 – 15.0)*	-	-	1.8 *(0.4 – 2.3)*	-	-
P. porpita	-	-	-	-	-	4.0 *(1.9 – 4.9)*	-	-
Crabs	8.2 *(3.0 – 11.7)*	0.8 *(0.4 – 1.8)*	-	-	-	6.5 *(1.7 – 18.0)*	3.5 *(2.8 – 4.3)*	5.5 *(4.8 – 6.3)*
Squid	-	-	-	0.3 *(0.3 – 0.3)*	5.0 *(4.5 – 5.5)*	8.0 *(4.7 – 70.5)*	0.6 *(0.5 – 2.0)*	0.3 *(0.2 – 1.2)*
Euphausiids & Shrimp	0.7 *(0.4 – 1.1)*	0.5 *(0.03 – 1.5)*	-	-	1.0 *(0.2 – 4.0)*	0.6 *(0.3 – 1.0)*	1.7 *(0.07 – 1.7)*	2.0 *(1.0 – 3.3)*
Fish	23.7 *(6.0 – 52.0)*	-	5.4 *(3.7 – 9.6)*	-	-	4.0 *(0.6 – 9.0)*	2.8 *(1.8 – 3.9)*	2.5 *(1.8 – 3.0)*
Copepods	0.8 *(0.4 – 1.0)*	3e-5 *(2e-5 – 0.002)*	2.0 *(0.8 – 17.0)*	0.002 *(0.001 – 0.1)*	0.02 *(0.02 – 0.02)*	0.6 *(0.2 – 0.9)*	0.2 *(0.02 – 0.4)*	0.3 *(0.1 – 1.0)*
Amphipods	11.1 *(7.8 – 13.6)*	0.1 *(0.06 – 0.3)*	1.0 *(0.9 – 1.2)*	0.2 *(0.2 – 0.6)*	1.0 *(0.4 – 4.0)*	2.3 *(1.2 – 13.3)*	1.0 *(0.3 – 1.7)*	2.0 *(0.5 – 3.6)*
Pteropods	-	0.008 *(0.007 – 0.009)*	4.2 *(4.0 – 8.7)*	0.8 *(0.8 – 0.9)*	0.1 *(0.1 – 0.3)*	3.8 *(2.0 – 11.0)*	0.4 *(0.2 – 0.7)*	2.0 *(1.2 – 3.0)*
Isopods	1.3 *(1.1 – 5.0)*	1.0*	21.0 *(14.0 – 28.0)*	-	-	4.0 *(3.3 – 4.5)*	-	-
Heteropods	-	-	-	2.0*	-	9.0 *(0.8 – 10.0)*	1.9 *(0.9 – 4.0)*	1.5 *(1.3 – 1.8)*
Chaetognaths	0.4 *(0.3 – 0.7)*	0.6*	-	0.5*	0.4 *(0.3 -0.6)*	2.7 *(1.9 – 11.5)*	1.0 *(0.5 – 2.0)*	0.6 *(0.4 – 0.8)*
Siphonophores	-	-	-	-	0.6 *(0.4 – 1.1)*	2.0 *(1.3 – 3.2)*	0.7 *(0.6 – 0.8)*	0.8 *(0.4 – 1.3)*
Salps	10.0*	0.5 *(0.3 – 0.5)*	2.2 *(1.3 – 3.1)*	-	2.5 *(1.8 – 3.3)*	7.0 *(4.0 – 11.0)*	2.0*	0.5*
Foraminifera	-	0.001 *(9e-4 – 0.02)*	-	0.1 *(0.1 – 0.1)*	0.1 *(0.1 – 0.6)*	-	0.5 *(0.4 – 0.8)*	0.5 *(0.4 – 1.0)*
**All**	**0.25** *(0.19 – 0.54)*	**3.1e-5** *(2.5e-5 – 6.4e-4)*	**0.98** *(0.24 – 2.6)*	**0.002** *(0.001 – 0.02)*	**0.014** *(0.01 – 0.02)*	**0.16** *(0.05 – 0.25)*	**0.05** *(0.01 – 0.15)*	**0.13** *(0.05 – 0.17)*

* Based on one value only

Supplementary Table S4 | Average weight per plastic particle. Values are expressed in mg and derived by dividing the total weight for each type and corresponding size class by the total number of respective particles. Note that no P- and F-type particles were found in the two largest size fractions (i.e., 0.15 – 1.5 cm and 1.5 – 5.0 cm).

**[mg]**	**0.05 – 0.15 cm**				**0.15 – 0.5 cm**				**0.15 – 1.5 cm**		**1.5 – 5.0 cm**	
	**H**	**N**	**P**	**F**	**H**	**N**	**P**	**F**	**H**	**N**	**H**	**N**
**Manta**												
**Average**	**0.51**			**0.20**	**2.04**	**5.97**	**5.50**	**0.32**	**6.96**	**61.28**	**87.60**	**1.69**
Min	0.02			0.20	0.10	0.10	5.50	0.10	0.25	0.05	1.70	0.40
Max	3.60			0.20	10.60	11.30	5.50	0.60	26.30	302.60	253.50	3.60
**Multinet**												
**Average**	**0.24**	**0.14**			**0.51**	**6.44**		**0.10**		**1.28**		**2.87**
Min	0.05	0.10			0.20	0.08		0.10		1.00		1.00
Max	1.17	0.20			1.00	107.63		0.10		1.90		3.90

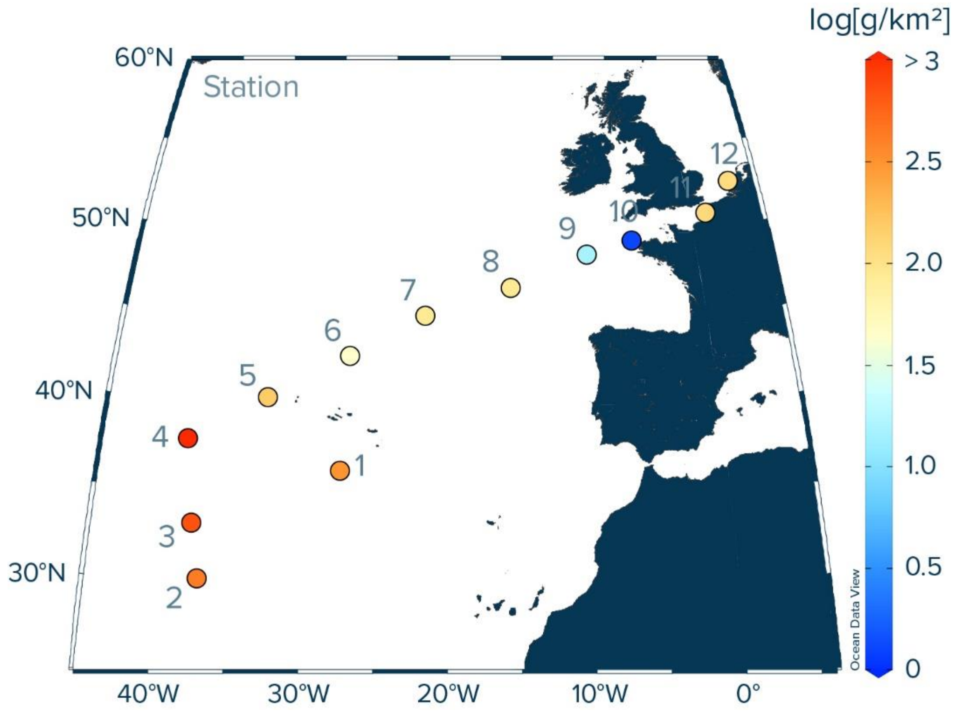

Figure S1 | Study sites located in the eastern North Atlantic Ocean and associated measured mass concentrations of floating plastic debris [g/km^2] at the ocean surface (debris size: 500 µm to 5 cm in size). The numbers 1-12 correspond to the station numbers. Note that the numerical concentrations shown here represent average values of three Manta trawl deployments per station. All values were corrected for wind-induced mixing as described by [1], [4] and [5]. The map was created using Ocean Data View (version 5.5.2; https://odv.awi.de/).

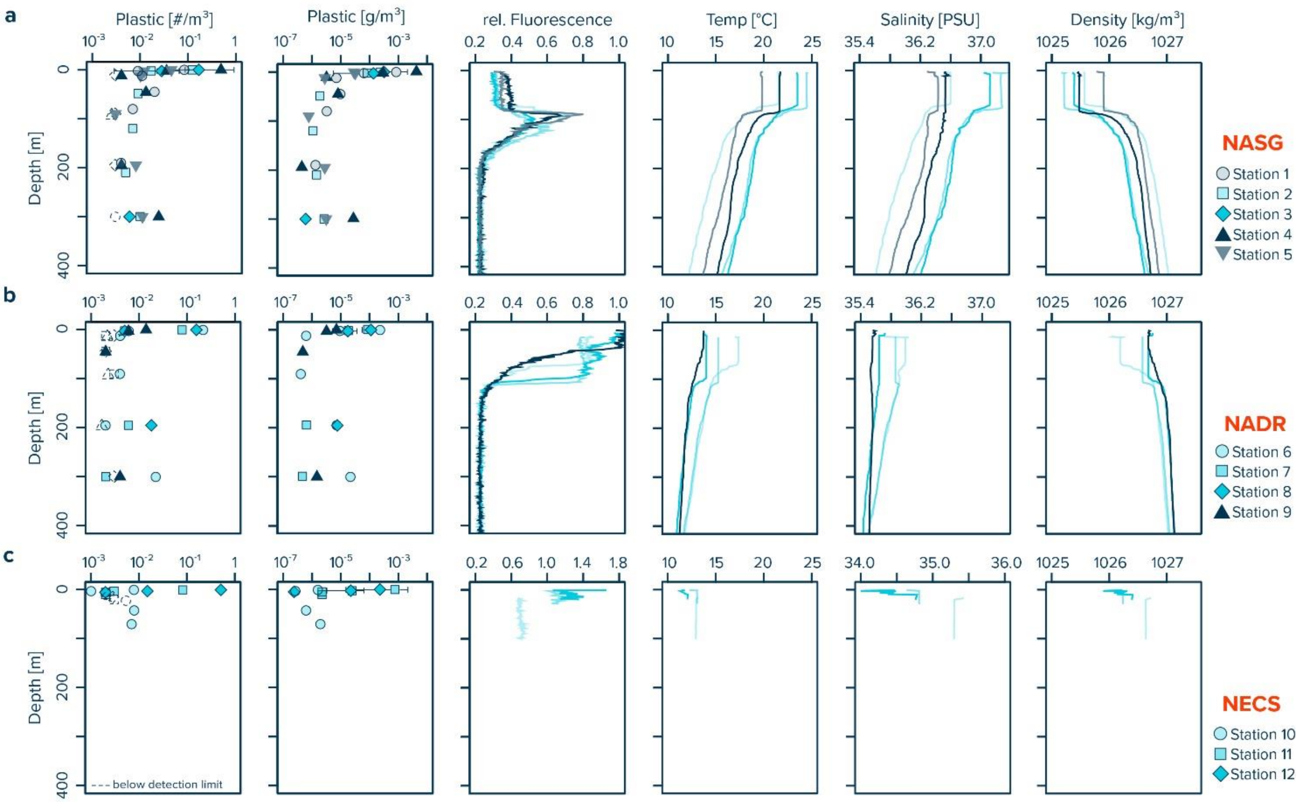

Figure S2 | Water column profiles of plastic (0.05 – 5 cm in size), chlorophyll fluorescence, temperature, salinity and density at for stations in (a) the North Atlantic Subtropical Gyre (NASG), (b) the North Atlantic Drift Region (NADR), and (c) the Northeast Atlantic Continental Shelves (NECS).

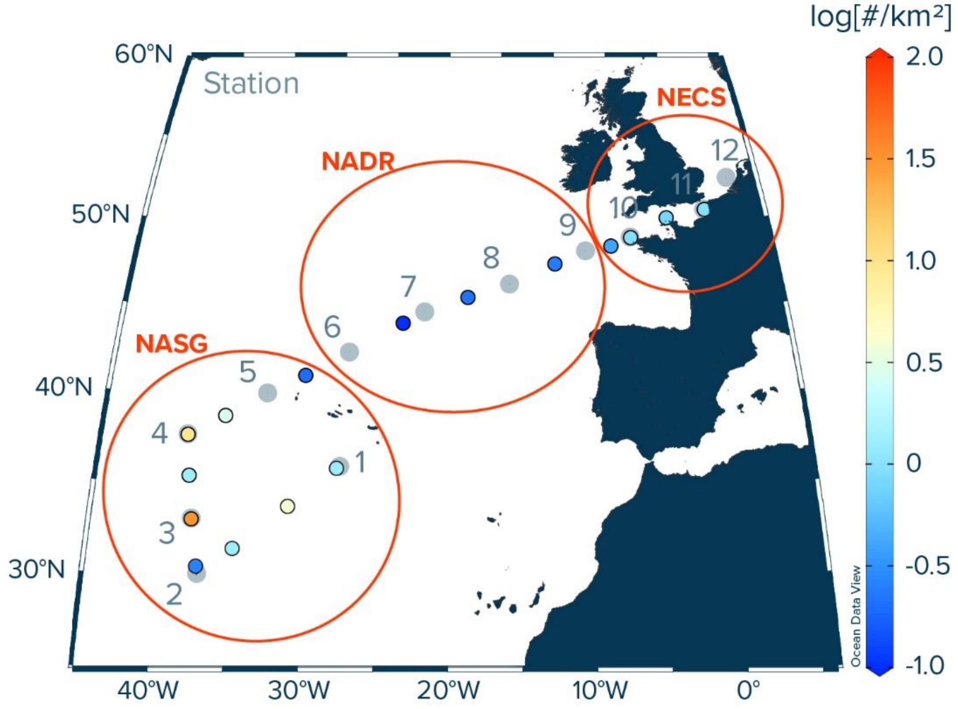

Figure S3 | Concentrations of floating megaplastic debris (> 50 cm) as derived using footage from a vessel-mounted GoPro in combination with the object-detection model developed by [6]. Grey dots and numbers 1-12 correspond to the trawling stations. NASG = North Atlantic Subtropical Gyre, NADR = North Atlantic Drift Region, NECS = Northeast Atlantic Continental Shelves. The map was created using Ocean Data View (version 5.5.2; https://odv.awi.de/).

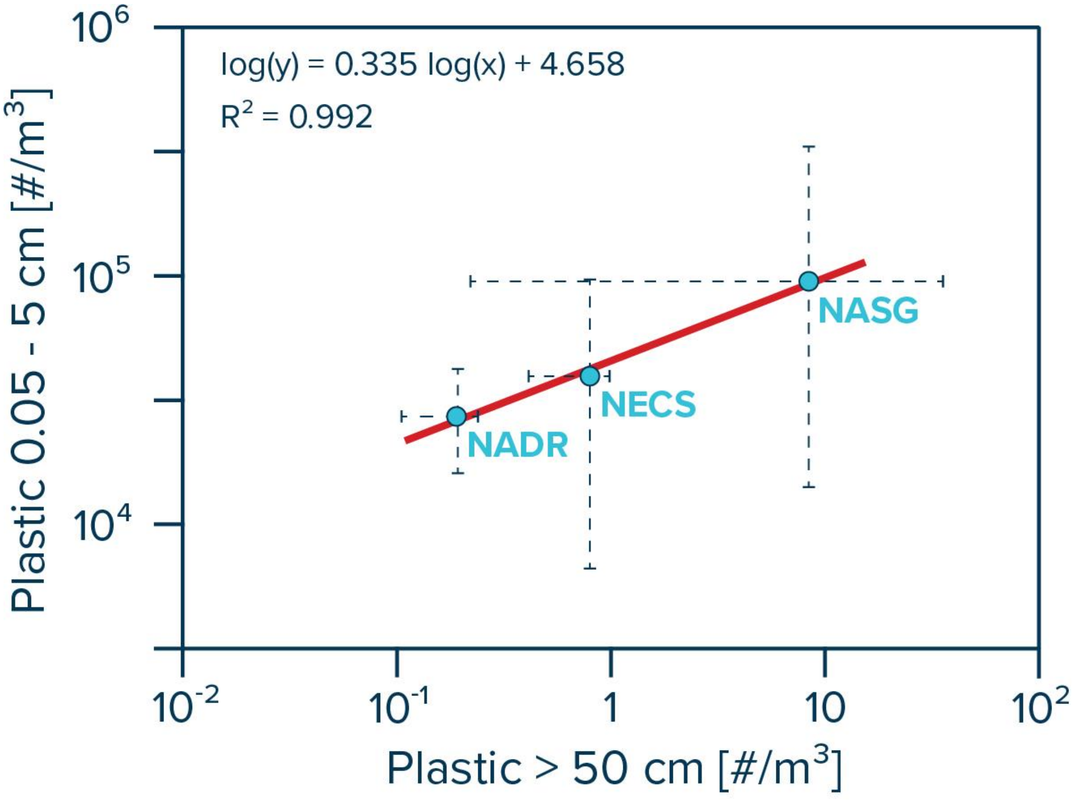

Figure S4 | Comparison between floating micro- and mesoplastic concentrations derived by Manta trawling (i.e., 0.05 – 5 cm in size) and concentrations of floating megaplastic debris (i.e., > 50 cm) derived by analyzing footage collected by a vessel-mounted GoPro with the object-detection model developed by [6]. Blue dots represent average values, with corresponding whisker extending to the smallest and largest concentrations measured. NASG = North Atlantic Subtropical Gyre, NADR = North Atlantic Drift Region, NECS = Northeast Atlantic Continental Shelves

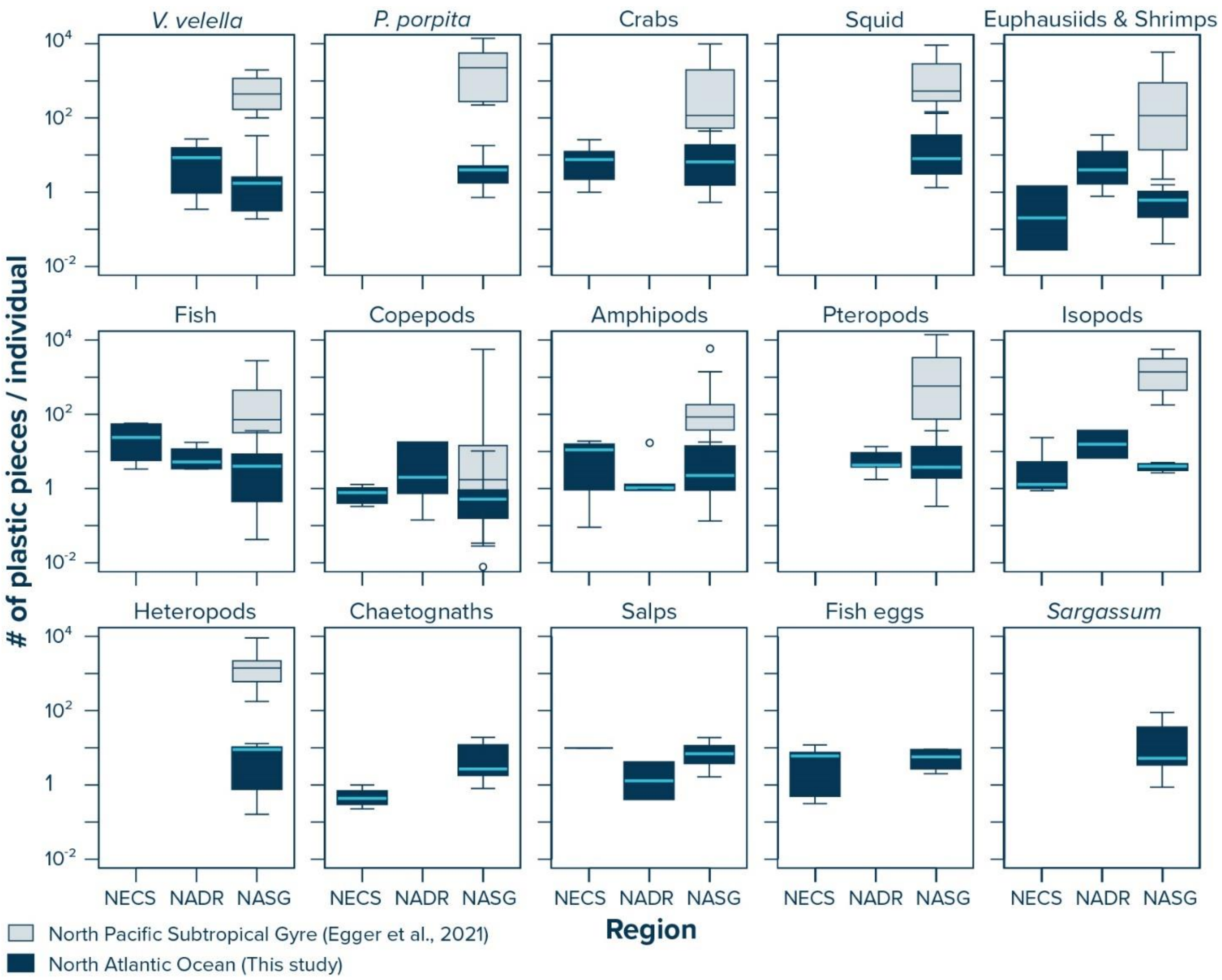

Figure S5 | Numbers of plastic pieces (0.05–5 cm in size) present per individual neuston taxon observed in Manta trawls deployed in the Northeast Atlantic Continental Shelves (NECS), the North Atlantic Drift Region (NADR), and the North Atlantic Subtropical Gyre (NASG). Horizontal lines represent median values. Box plots extend from the 25th to the 75th percentiles, while whiskers extend from the minimum to the maximum observed values. Dots indicate outliers. Note that two outliers fall outside of the boundaries of the y-axis depicted here, with values of 0.000361 #/individual Copepods in the NECS and 0.00110 #/individual Amphipod in the NADR. All ratios are based on uncorrected plastic concentrations (i.e., no correction for wind-induced mixing) to allow for comparisons of equivalent measures (i.e., only comparing what was caught in the Manta trawl in particular conditions).

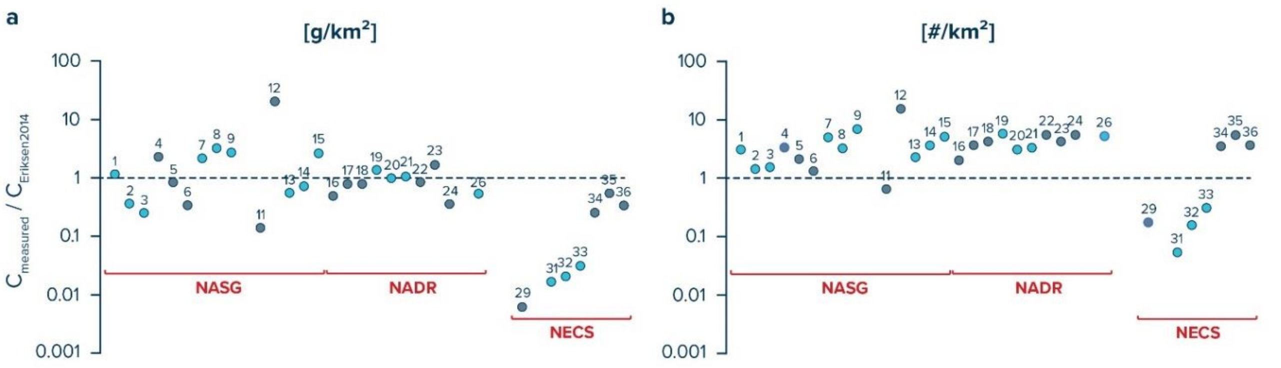

Figure S6 | Comparison between (a) mass and (b) numerical concentrations of floating microplastics measured in this study (particle size range: 500 µm – 5 mm) vs. predicted by Eriksen and co-workers [7] (particle size range: 330 µm – 4.75 mm). Values from [7] were extracted for each Manta trawl location using ArcGIS. Dot numbers correspond to the respective Manta trawl number. NASG = North Atlantic Subtropical Gyre (Manta trawl #1 – 15), NADR = North Atlantic Drift Region (Manta trawl #16 – 26), NECS = Northeast Atlantic Continental Shelves (Manta trawl #29 – 36). Dashed horizontal lines indicate a perfect fit between observed and predicted concentrations. Note that no plastic particles > 500 µm were observed in Manta trawl #10, 25, 27, 28, and 30.

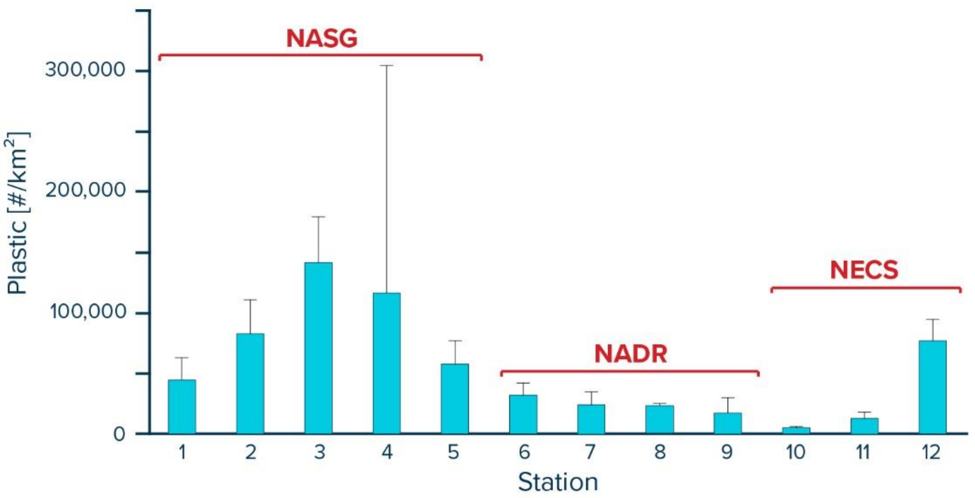

Figure S7 | Variability of floating plastic concentrations (0.05 – 5 cm in size) measured in the three consecutive Manta trawl deployments at our study sites (average values and standard deviation are depicted). NASG = North Atlantic Subtropical Gyre, NADR = North Atlantic Drift Region, NECS = Northeast Atlantic Continental Shelves

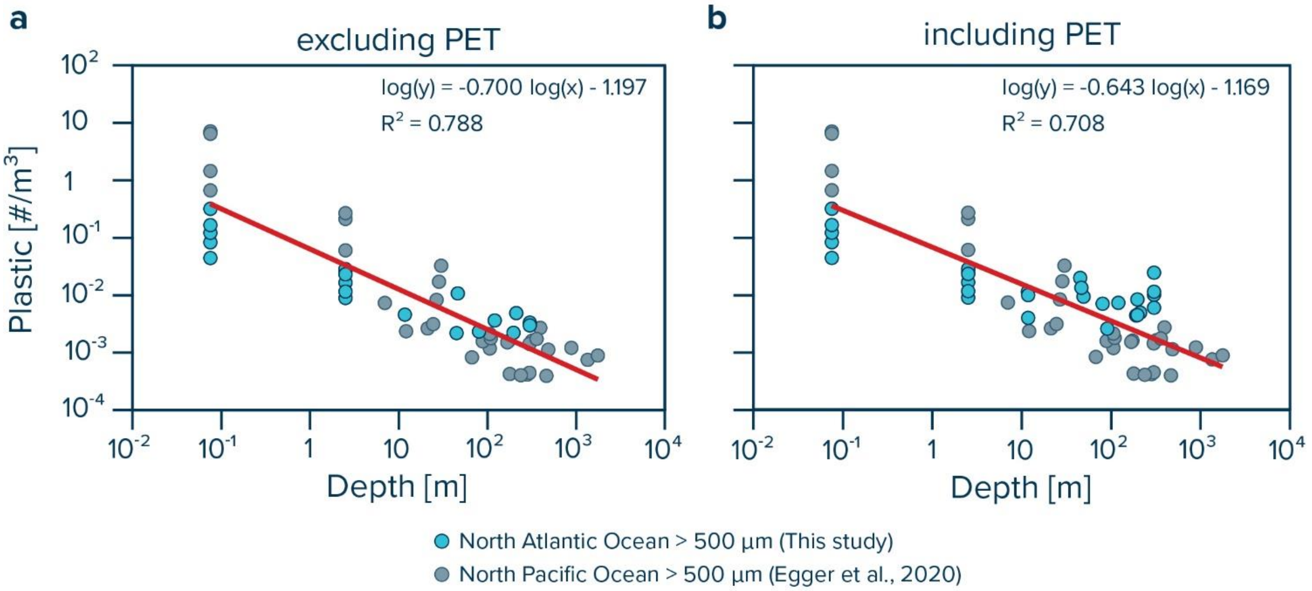

Figure S8 | log-log plots of water depth vs. numerical concentrations of plastic debris between 0.05 – 5 cm in size. The red line represents the log-log linear regression fit. Correlations are presented for plastic concentrations derived by (a) excluding and (b) including PET fragments in this study (blue dots). Grey dots represent data from the North Pacific subtropical gyre [8].

References

1. Kukulka, T., Proskurowski, G., Morét-Ferguson, S., Meyer, D. W. & Law, K. L. The effect of wind mixing on the vertical distribution of buoyant plastic debris. *Geophysical Research Letters* **39**, 1–6 (2012).

2. Reisser, J. *et al.* The vertical distribution of buoyant plastics at sea: an observational study in the North Atlantic Gyre. *Biogeosciences* **12**, 1249–1256 (2015).

3. Kooi, M. *et al.* The effect of particle properties on the depth profile of buoyant plastics in the ocean. *Scientific Reports* **6**, 1–10 (2016).

4. Lebreton, L. *et al.* Evidence that the Great Pacific Garbage Patch is rapidly accumulating plastic. *Scientific Reports* **8**, 1–15 (2018).

5. Egger, M. *et al.* A spatially variable scarcity of floating microplastics in the eastern North Pacific Ocean. *Environmental Research Letters* **15**, 114056 (2020).

6. de Vries, R., Egger, M., Mani, T. & Lebreton, L. Quantifying floating plastic debris at sea using vessel-based optical data and artificial intelligence. *Remote Sensing* **13**, 1–16 (2021).

7. Eriksen, M. *et al.* Plastic pollution in the world's cceans: more than 5 trillion plastic pieces weighing over 250,000 tons afloat at sea. *PLoS ONE* **9**, e111913 (2014).

8. Egger, M., Sulu-Gambari, F. & Lebreton, L. First evidence of plastic fallout from the North Pacific Garbage Patch. *Sci Rep* **10**, 7495 (2020).
